# Neuroprotective Effect of *Ixeris dentata* Extract on Trimethyltin-Induced Memory Impairment in Rats

**DOI:** 10.3390/cimb46110699

**Published:** 2024-10-22

**Authors:** Minsook Ye, Daehyuk Jang, Sun-young Lee, Kyu-Ri Kim, Sung Ja Rhie, Jin Kyung Oh, Insop Shim

**Affiliations:** 1 Department of Physiology, College of Medicine, Kyung Hee University, Seoul 02435, Republic of Korea; jh486ms22@naver.com (M.Y.); nisclist@nate.com (D.J.); tramp68@naver.com (S.-y.L.); kyuri_kim@khu.ac.kr (K.-R.K.); 2 Department of Beauty and Health, Halla University, Wonju-si 26404, Republic of Korea; sjlee@halla.ac.kr; 3 APK Science, 16-6, Pyeongchang 12-gil, Jongno-gu, Seoul 03009, Republic of Korea; eireneuni@hanmail.net

**Keywords:** *Ixeris dentata* (ID), trimethyltin (TMT), learning and memory, morris water maze (MWM), cAMP-response element-binding protein (CREB)

## Abstract

Alzheimer’s disease (AD) is a representative neurodegenerative disease characterized by the structural and functional degeneration of neurons. The present study investigated the neuroprotective effect of *Ixeris dentata* (ID) extract on trimethyltin (TMT)-induced memory deficit in the rat. Cognitive improving effect and neuronal activity of ID were assessed by using Morris water maze (MWM) test and choline acetyltransferase (ChAT), cAMP-response element-binding protein (CREB) immunohistochemistry. Seven days after TMT injection (8.0 mg/kg, i.p.), each group of rats was administered saline, water extract of ID (WID, 400 or 800 mg/kg, p.o.), ethanol extract of ID (EID, 400 or 800 mg/kg, p.o.), or caffeic acid (CAF, 30 mg/kg, i.p.) daily for fourteen days. **Results**: Treatment with EID and CAF produced a significant improvement in escape latency time of the acquisition, and retention time in the target area of the MWM task. Additionally, administration of EID or CAF markedly alleviated TMT-induced loss of ChAT- and CREB-immunoreactive cells in the hippocampus. The results demonstrated that EID has a protective effect against TMT-induced memory deficit, partly through increasing the CREB and cholinergic signaling pathway in the hippocampus. These results suggest that ethanol extracts of ID might be useful for improving cognitive functions in neurodegenerative diseases such as Alzheimer’s disease.

## 1. Introduction

Neurodegenerative diseases are characterized by the progressive loss of neurons, a process known as neurodegeneration. These disorders are characterized by the progressive degeneration of neurons, which leads to cognitive decline and other functional impairments. In addition to AD, other common neurodegenerative diseases include Parkinson’s disease, amyotrophic lateral sclerosis (ALS), Huntington’s disease, and multiple sclerosis. AD, the most common type of dementia, is a neurodegenerative disorder characterized by memory impairment, cognitive malfunction, and character change [1]. The number of patients and social costs associated with dementia or AD have been increasing consistently and many research studies for diagnosis and treatment of AD have been conducted through in vitro and in vivo studies [2,3]. Several animal models of memory deficit have been reported to find a successful treatment for AD. Trimethyltin (TMT) intoxication is widely recognized as a suitable model for chronic neuronal degeneration associated with cognitive impairment, making it a valuable tool for studying AD [4]. TMT is a neurotoxic compound which generates deterioration of neurons in the limbic system, especially in the hippocampus [5,6]. The exposure to TMT results in necrosis of hippocampal pyramidal and granule cells, leading to disruptions in normal behavioral patterns, alterations in hippocampal physiological activity, and changes in neurochemical markers associated with endogenous hippocampal neurotransmitters. In rodents, TMT leads to loss of the hippocampus due to degeneration of pyramidal neurons in the hippocampus and the hippocampus-associated area [7,8]. Selective neuronal lesioning by TMT injection triggers abnormal behavior and cognitive impairments such as aggressiveness, seizures, and memory deficit [9].

*Ixeris dentata* (ID) has been used as a traditional herbal medicine in East Asian countries for treatment of mithridatism, calculus, and tumors [10,11]. Several studies revealed that ID extract has antimutagenic and antioxidant effects, leading to the suppression of cancer cell growth and induction of tumor necrosis [10]. It has been reported that ID has an anti-inflammatory effect on skin cells [12] and an antiallergic effect [11]. Furthermore, extract of ID has been shown to have a neuroprotective effect against oxidative stress by kainic acid in the brain of mice [10]. Although the ID has been studied to treat several diseases, the cognitive improvement effect of ID has not been reported yet and underlying neural mechanisms are barely understood.

Caffeic acid (CAF), one of the major hydroxycinnamic acids, is an organic compound found in ID, coffee, and herbs like thyme and rosemary [13,14]. It has been shown that CAF improves cognitive function by reducing neurodegeneration in animals with chloride-induced dementia [15]. CAF has also been reported to have a neuroprotective effect by preventing oxidative stress-induced neurodegeneration in animal models of AD [16,17]. These results revealed that CAF may alleviate neurodegeneration in AD animal models. It is possible that CAF, one of the major bioactive components in ID, may have an active role in mediating a pharmacological effect of ID [11].

Choline acetyltransferase (ChAT) is an enzyme responsible for the biosynthesis of the neurotransmitter acetylcholine, and its activity serves as a well-known marker of central cholinergic neurons. Notably, there is a negative correlation between ChAT activity and the severity of dementia [18]. Degeneration of the central cholinergic system, as indicated by decreased ChAT activity, is a characteristic feature of AD, including dementia [19]. Furthermore, ChAT levels have been positively correlated with improved mental functions in AD patients [20].

The cAMP-response element-binding protein (CREB) is a cellular transcription factor that binds to particular DNA sites to regulate transcription. CREB is also a component of intracellular signaling that regulates a variety of biological functions including circadian rhythm and memory. CREB is involved in an essential step to convert short-term to long-term memory in the hippocampus [21]. It has been reported that inhibition of CREB in the CA1 of the hippocampus disturbs forms of synaptic plasticity and spatial learning [22,23].

This study aimed to evaluate the neuroprotective effect of ID extract on TMT-induced learning and memory deficits in rats. Spatial learning and memory were assessed using the Morris water maze test. The expression of ChAT and CREB, markers of hippocampal neuronal activity, was investigated through immunohistochemistry.

## 2. Materials and Methods

### 2.1. Regents

ID was purchased from the Jeong Dong Myong Sseumbagwi (Iksan, Republic of Korea) company. The ID (300 g) was extracted in two different ways. One was boiled in distilled water at 100 °C for 2 h and the other was boiled twice in 70% ethanol at 60 °C for 2 h. Each of the extracts was filtered through Whatman No. 1 filter using a vacuum pump and was condensed using a rotary vacuum evaporator (N-1000S; EYELA, Tokyo, Japan). The concentrates were frozen and dried with a freeze dryer (Eyela FD-800; Tokyo Rikakikai Co., Tokyo, Japan). The yields of water extract (defined as WID), and ethanol extract (defined as EID) of ID were 22.7% and 19.5%, respectively. CAF was purchased from Sigma (St. Louis, MO, USA).

**Figure 1:**
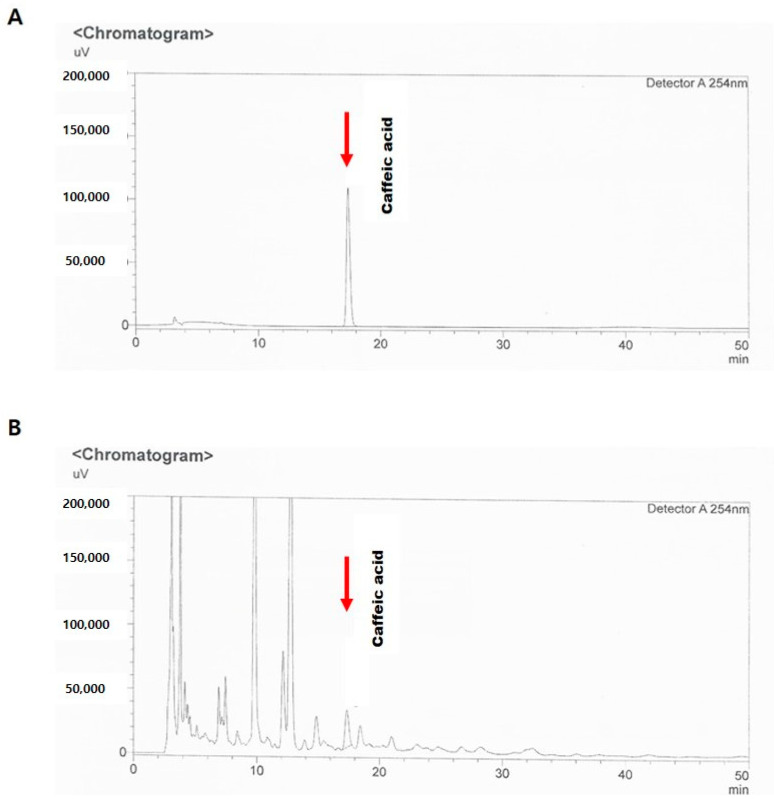
**HPLC analysis of the standard material to IXD.** CAF was utilized as an authentic standard (arrows) and the concentrations of CAF in WIXD (**A**) and CAF in EIXD (**B**) were found.

For the phytochemical analysis of EID, high-performance liquid chromatography (HPLC) was performed. The EID was accurately weighed to 100 mg and then dissolved in 10 mL 100% methanol. The sample was extracted in an ultrasonic bath for 60 min at 30 °C. The suspension was filtered, and the filtrate was evaporated in vacuo. The residue was dissolved in methanol (10 µL), and the obtained solution was filtered through a membrane filter (5 µm pore size) prior to injection. For the quantitative analysis of EID, CAF, one of the known polyphenol constituents of EID, was diluted in acetonitrile. The solution was filtered through a membrane filter (5 µm pore size) prior to injection. The HPLC analysis was performed using Gilson system equipped with an in-line degasser and a spectrophotometer detector. The UV spectra were collected across the range of 254 to 280 nm and monitored at 254 nm for chromatograms. Empower software 3 was used for instrument control, data collection, and data processing. The HPLC column was an Inspire C18 (4.6 × 250 mm, 5 µm). The mobile phase was an isocratic system with acetonitrile (A) glacial acetic acid (B) run for 15 min. The ratio of A : B was 89 : 11. The flow rate was 1 mL/min. The injection volume for all samples and standard solutions was 10 µL. Using this method, the concentration of CAF in EID was calculated to be 4 µg/g as described in Figure 1.

### 2.2. Animals

The male Sprague-Dawley rats weighing 250–270 g were purchased from the Samtako Animal Co. (Seoul, Republic of Korea). The animals were allowed to acclimatize themselves for at least 7 days prior to the experimentation. The rats were housed in individual cages under controlled conditions: 12 h light/dark cycle (lights on at 8:00 and off at 20:00) and at 24 °C room temperature. Food and water were available ad libitum. These experiments were executed in accordance with the National Institutes of Health Guide for the Care and Use of Laboratory Animals, revised in 1996, and were approved by the Institutional Animal Care and Use Committee of Kyung Hee University (KHUAP(SE)-13-041). 

### 2.3. Experimental Groups

**Figure 2:**
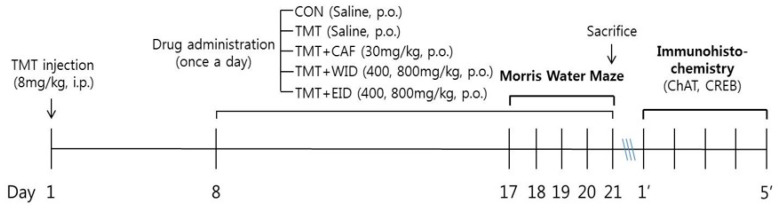
Experimental schedule of this study.

The rats received intraperitoneal (i.p.) injection of trimethyltin (TMT, 8.0 mg/kg, Sigma, St Louis, MO, USA) dissolved in physiological saline solution, and were returned to cages. From the 8th day after the injection of TMT, the animals were randomly divided into Con (n = 7), TMT (n = 7), CAF 30 (CAF 30 mg/kg, n = 6), WID 400 (n = 6) and 800 (n = 6) (WID 400 and 800 mg/kg), and EID 400 (n = 7) and 800 (n = 7) (EID 400 and 800 mg/kg) treated groups. CON group was added as a non-treated and naïve normal group. CAF was used as a positive control. CAF, WID, and EID were dissolved in saline and orally administered for 2 weeks. From the 17th day, the water maze task was performed for 5 days. The experimental schedule is shown in Figure 2.

### 2.4. Morris Water Maze

The MWM test was performed in a circular pool that was 200 cm in diameter and 35 cm deep. The pool was filled with water that was maintained at 22  ±  2 °C temperature and contained powdered skim milk to make it opaque. A platform (0.15 m in diameter, 0.2 m in height) was placed within the pool and top surface of platform was fixed at 1.5 cm below the surface of the water. Several visual cues were placed around the pool in plain sight of the animals. The pool was divided into four quadrants of equal area and each trial was initiated at one of the different positions.

A digital camera was connected to a personal computer with a computerized recording system (S-MART; Panlab Co., Barcelona, Spain) and was mounted to the ceiling above the center of the pool. The top, neck, and back of the rat were dyed (non-toxic) black (ReEn secret for black hair foam type, LG Life and Health, Seoul, Republic of Korea) in order to facilitate rat tracking in the water maze. Rats were tested with three trials of the acquisition test per day for 4 consecutive days and received the retention test on the 5th day. During the acquisition test, the animals were allowed to train to find the hidden platform for a maximum of 180 s at each trial. The latency to escape onto the platform was recorded. For the retention test, they received a minute probe trial in which the platform was removed from the pool. The performance of the test animals was assessed by a program for behavioral analysis (S-MART; Panlab Co., Barcelona, Spain) in each trial. The retention was measured by the percentage of time spent searching for the platform.

### 2.5. Immunohistochemistry

After the performance of behavioral tasks, the rats were anesthetized with sodium pentobarbital (80 mg/kg, IP) and were perfused through with normal saline followed by 4% paraformaldehyde (Sigma, St Louis, MO, USA). The brains were post-fixed in the same fixative for 24 h and cryoprotected in PBS with 20% sucrose at 4 °C. Coronal sections were serially cut into 30 μm thicknesses using a cryostat microtome (Leica CM1850; Leica Microsystems Ltd., Nussloch, Germany) and stored at 4 °C in cryoprotectant containing PBS and 20% sucrose. Before starting the immunohistochemistry, the sections were washed by PBS containing 0.2% Triton X-100 (PBST) three times.

The primary antibodies used the following specific antigen: CREB (rabbit polyclonal CREB; 1:1,000 dilution, Cell signaling, Boston, MA, USA). The prepared primary antibody was mixed with 0.2% PBST, 2% blocking serum (Sigma, St Louis, MO, USA). The sections were incubated in the primary antiserum for 72 h at 4 °C. After three rinses with PBST, the sections were incubated with secondary antibody diluted 1:1,000 in PBST (Vector Laboratories Co., Burlingame, CA, USA) for 2 h at room temperature. Following a further rinsing in PBST, the sections were incubated in avidin-biotin-peroxidase complex reagent (Vector Laboratories, Burlingame, CA, USA) for 2 h at room temperature. The sections were washed in PBST; those were developed using diaminobenzidine peroxidase substrate kit (Vector Laboratories, Burlingame, CA, USA) as the chromogen.

The images were captured using a DP2-BSW imaging system (Olympus, San Jose, CA, USA) and they were processed using Adobe Photoshop (Adobe Systems Inc., San Jose, CA, USA). For measuring the cells that were positive for CREB, the grid was placed on CA1 and CA3 of the hippocampus according to the method of Paxinos et al. (1985). The number of cells was counted at 200× (CREB) magnification using a microscope rectangle grid that measured 200 × 200 mm. The cells were counted in three sections per rat within the hippocampal CA1 and CA3 areas. The brain sections were visually inspected at 3 different anteroposterior levels extending from −2.12 to −6.04 mm [24].

### 2.6. Statistical Analysis

All statistical analyses were performed using IBM SPSS Statistics 23.0. The sample sizes for each group were as follows: control group (N = 6), TMT group (N = 6), TMT + CAF 30 (N = 6), TMT + WID 400 (N = 7), TMT + WID 800 (N = 7), TMT + EID 400 (N = 8), and TMT + EID 800 (N = 8). Prior to conducting one-way ANOVA, the normality of the data distribution was evaluated using the Shapiro–Wilk test (*p* > 0.05), and the homogeneity of variances was confirmed using Levene’s test (*p* > 0.05). Once the assumptions of normality and homogeneity of variances were satisfied, one-way ANOVA was applied to assess group differences, followed by the LSD post-hoc test for pairwise comparisons. Data are presented as mean ± standard error of the mean (SEM), and statistical significance was set at *p* < 0.05.

## 3. Results

### 3.1. Effect of ID Extracts on TMT-Induced Memory Deficits in the Morris Water Maze Test

**Figure 3:**
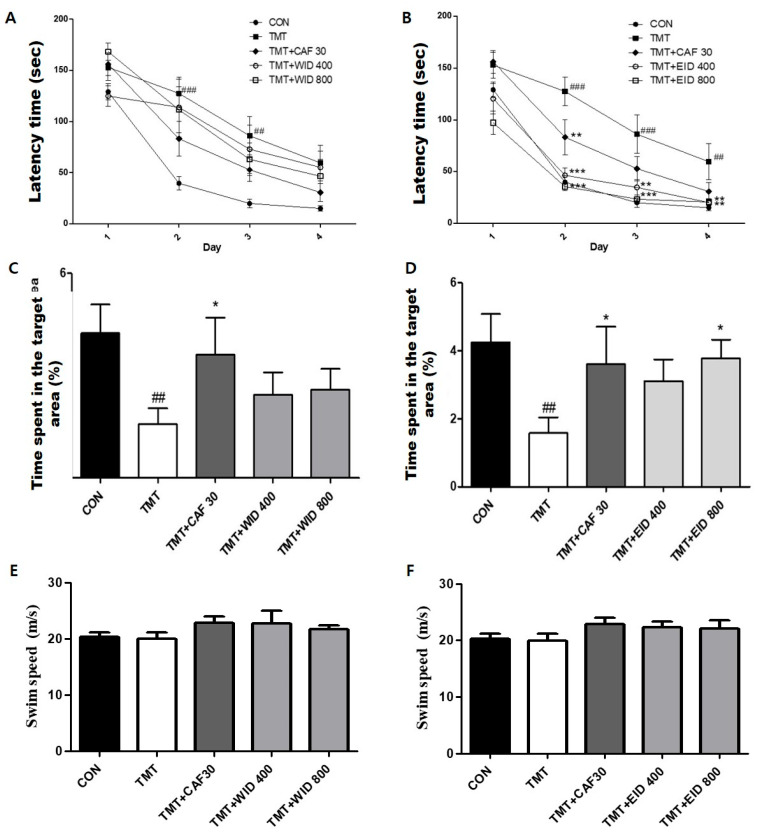
**Effects of WID and EID administration on acquisition test in the MWM task.** The latency time of WID (**A**) and EID (**B**) administration groups was compared with TMT-induced memory-deficit rats on acquisition task. The percentage of retention time without a platform of WID (**C**) and EID (**D**) administration compared with TMT-induced memory-deficit rats on the retention in the MWM test. The swim speed of WID (**E**) and EID (**F**) administration compared with TMT-induced memory-deficit rats on the retention in the MWM test. ##, *p* < 0.01 vs. CON, ###, *p* < 0.001 vs. CON, *, *p* < 0.05 vs. TMT. **, *p* < 0.01 vs. TMT, ***, *p* < 0.001 vs. TMT.

The effect of the CAF, WIDs (400 and 800 mg/kg), and EIDs (400 and 800 mg/kg) on spatial learning was evaluated on the Morris water maze (MWM) test. The CON group rapidly learned it and the TMT group showed a worse performance than other groups in the MWM acquisition test (*p* < 0.001 on Day 2, *p* < 0.01 on Day 3, as shown in Figure 3A; and *p* < 0.001 on Day 2 and 3 and *p* < 0.01 on Day 4, respectively, as shown in Figure 3B). The escape latency of the CAF30 and all WID groups was not significantly different from that of the TMT group in Figure 3A. As shown in Figure 3B, the escape latency of CAF 30 was significantly decreased (*p* < 0.01 on Day 2) compared with the TMT group. Treatment with EID (400 and 800 mg/kg) significantly reduced the escape latency in the acquisition test (EID 400, *p* < 0.001 on Day 2, *p* < 0.01 on Day 3 and 4; EID 800, *p* < 0.001 on Day 2 and 3, and *p* < 0.01 on Day 4, respectively).

The retention test was examined by analyzing the percentages of time on the position where the platform was expected to be (Figure 3C,D). The TMT group spent less time around the platform than the CON group on the retention test (*p* < 0.01). However, the CAF 30 and EID 800 groups spent significantly more time around the platform in the retention test (*p* < 0.05), as seen in Figure 3D. The TMT group was not significantly different from the other groups in mean swimming speed, as calculated by dividing the total swim distance by latency (*p* = 0.4991; Figure 3E), (*p* = 0.4034; Figure 3F). 

### 3.2. Effects of IXD Extracts on TMT-Induced Immunohistochemical Alterations of ChAT, CREB in the Hippocampus

**Figure 4:**
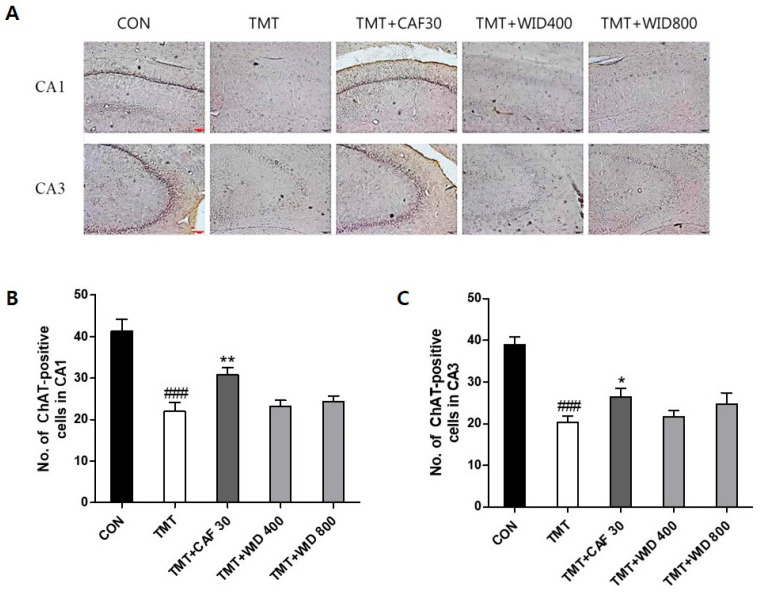
Immunohistochemical analysis of choline acetyltransferase (ChAT)-stained neurons in hippocampal areas after CAF and WID administrations. (**A**) Representative photographic showing distribution of ChAT-immunopositive cells on the hippocampal area (×100). Scale bar: 200 µm. Number of ChAT-immunopositive cells in CA1 (**B**) and CA3 (**C**) on the hippocampal areas. The values are presented as means ± S.E.M (n = 8). ###, *p* < 0.001 vs. CON, *, *p* < 0.05 vs. TMT, **, *p* < 0.01 vs. TMT.

**Figure 5:**
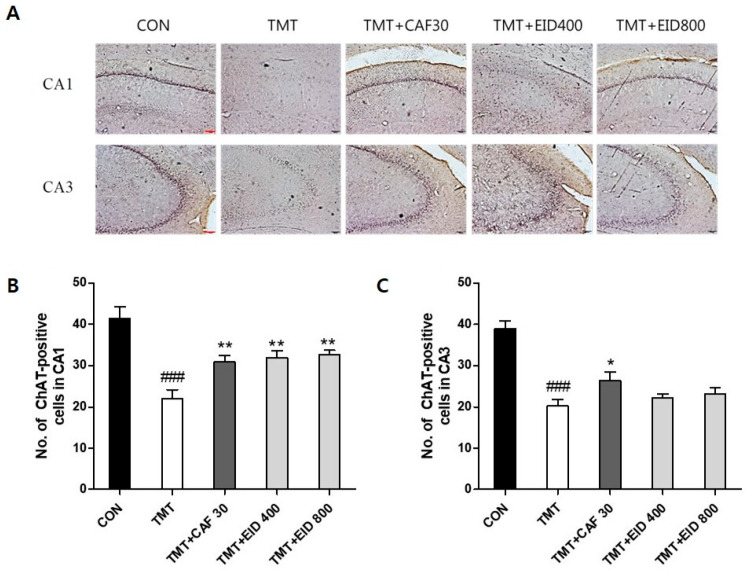
Immunohistochemical analysis of choline acetyltransferase (ChAT)-stained neurons in hippocampal areas after CAF and EID administrations. (**A**) Representative photographic showing distribution of ChAT-positive cell on the hippocampal area (×100). Scale bar: 200 µm. Number of ChAT-positive cells in CA1 (**B**) and CA3 (**C**) on the hippocampal areas. The values are presented as means ± S.E.M (n = 8). ###, *p* < 0.001 vs. CON, * *p* < 0.05, **, *p* < 0.01 vs. TMT.

**Figure 6:**
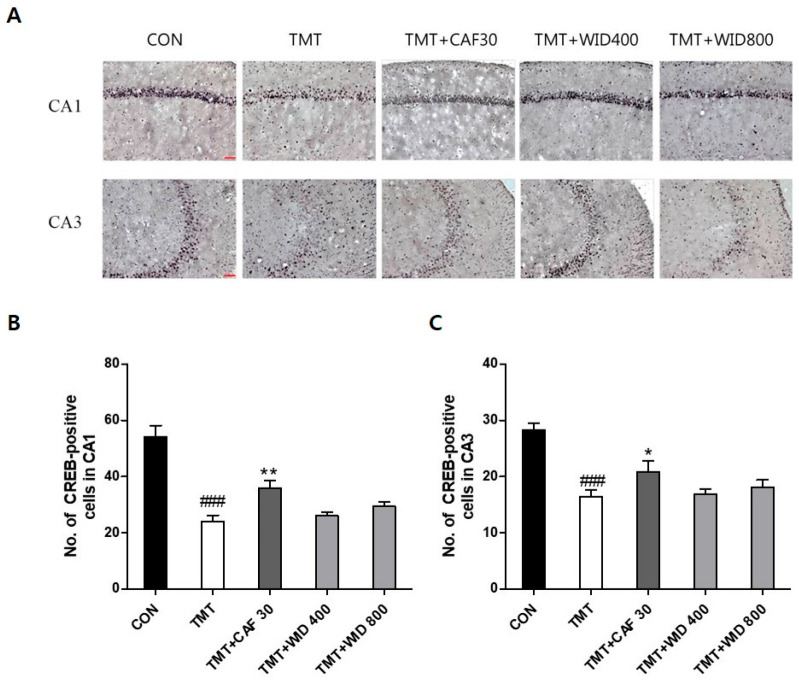
Immunohistochemical analysis of cAMP-response element-binding protein (CREB)-stained neurons in hippocampal areas after CAF and WID administrations. (**A**) Representative photographic showing distribution of CREB-positive cells on the hippocampal area (×200). Scale bar: 200 µm. Number of CREB-positive cells in CA1 (**B**) and CA3 (**C**) on the hippocampal areas. The values are presented as means ± S.E.M (n = 8). ###, *p* < 0.001 vs. CON, * *p* < 0.05, **, *p* < 0.01 vs. TMT.

**Figure 7:**
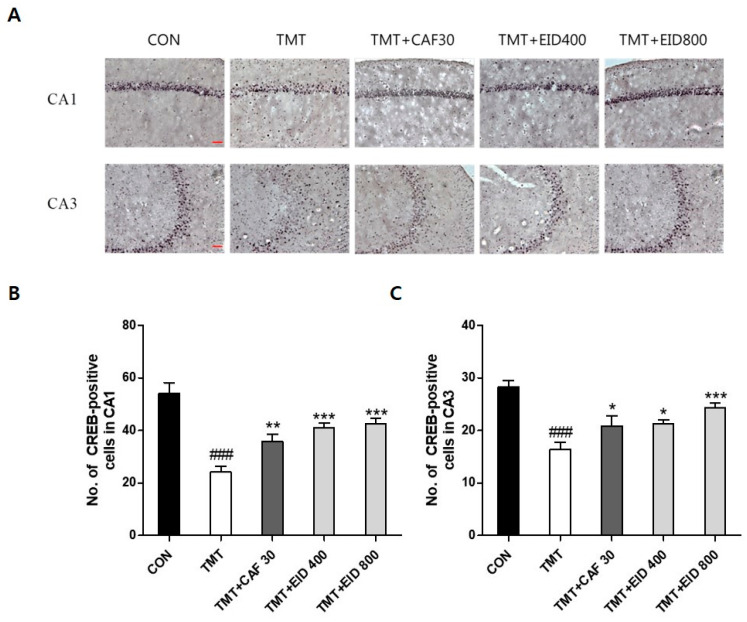
Immunohistochemical analysis of cAMP-response element-binding protein (CREB)-stained neurons in hippocampal areas after CAF and EID administrations. (**A**) Representative photographic showing distribution of CREB-positive cells on the hippocampal area (×200). Scale bar: 200 µm. Number of CREB-positive cells in CA1 (**B**) and CA3 (**C**) on the hippocampal areas. The values are presented as means ± S.E.M (n = 8). ###, *p* < 0.001 vs. CON, *, *p* < 0.05 vs. TMT, **, *p* < 0.01 vs. TMT, ***, *p* < 0.001 vs. TMT.

The results of ChAT- and CREB-positive cells in the hippocampus of the experimental groups are presented in Figure 4, Figure 5, Figure 6 and Figure 7. Compared to the CON group, ChAT (*p* < 0.001, Figure 4 and Figure 5) and CREB immunoreactivity (*p* < 0.001, Figure 6 and Figure 7) in the TMT group were significantly decreased in the hippocampus. However, compared to the TMT group, ChAT immunoreactivity in the CAF 30 group was significantly upregulated in the CA1 (*p* < 0.01, Figure 4) and CA3 regions (*p* < 0.05, Figure 5). Similarly, CREB-immunoreactive cells in the CAF 30 group were significantly upregulated in the CA1 (*p* < 0.01, Figure 6) and CA3 regions (*p* < 0.05, Figure 7) compared to the TMT group. Administration of EIXD also significantly increased the number of CREB- and ChAT-positive neurons in the hippocampus, similar to the effects observed in the CAF 30 group, while WIXD administration did not affect the immunoreactivity of ChAT and CREB. Specifically, EIXD 400 and 800 treatments significantly increased ChAT levels in the hippocampal CA1 area (*p* < 0.01, Figure 4), but not in the CA3 area (Figure 5). CREB-positive cells were significantly upregulated in the CA1 region in the EIXD 400 and 800 groups (*p* < 0.001, Figure 6) and in the CA3 region (*p* < 0.05 and *p* < 0.001, respectively, Figure 7).

## 4. Discussion

The present study demonstrated that TMT-induced memory deficits and neurodegeneration in the hippocampus were eased by treatment with IXD and its bioactive compound CAF. Treatment with EIXD and CAF lessened TMT-induced memory deficits and neurodegeneration in the MWM task, and had a neuroprotective effect against TMT-induced decreases in ChAT- and CREB-immunoreactive neurons.

ID is known to have antimutagenic, antioxidant, anti-inflammatory, and antiallergic effects, and various sesquiterpenes and phenol compounds have been isolated from ID [10,11,25,26]. Among these, CAF, one of the common phenolic acids, has been reported to improve cognitive function by reducing neurodegeneration in AD animal models [27,28]. Thus, we focused on ID and CAF and demonstrated both agents’ neuroprotective effect on TMT-induced learning and memory impairments.

The MWM test has evaluated learning and memory by spatial learning task, so has been used in the validation for cognitive impairment animal model [29]. Many studies have indicated that the TMT-induced memory deficit model impairs behavioral performance in tests such as the passive avoidance test and water maze task [30]. The performance of the MWM task with TMT-induced rats in the current study shows a similar output of spatial learning compared to previous studies. From immunohistochemical results, furthermore, EID and CAF were more effective in protecting hippocampal CA1 cells than CA3. Since it is known that TMT-induced cell death in the hippocampal CA1 area is closely correlated with behavioral deficits in learning and memory, and the hippocampal CA1 area plays an important role in memory processes involved in the Morris water maze, our results indicated that EID and CAF have a neuroprotective effect on preferentially hippocampal CA1 cells. Thus, our results suggest that ID extracts may alleviate memory deficit as an increase in spatial retention and may relate with long-term memory.

CREB is an important transcription factor of genes involved in growth, survival, and synaptic plasticity of neurons [31]. Many researchers have provided that CREB mutation is related with disruption of long-term memory or cognitive function deficit, and damaged or insufficient CREB leads to neurodegeneration in the hippocampal area, so CREB signaling has an important role for learning and memory [32]. Our results showed that EID significantly increased CREB levels in the CA1 and CA3 of the hippocampal area compared with TMT-induced rats. Thus, our results suggest that TMT-induced memory impairment may be closely related to CREB levels in the rat hippocampus. It means the TMT-induced downregulation of CREB causes memory loss in rats. From these results, we suggest that EID may relate to cholinergic function and a neuroprotective effect for improvement of learning and memory.

In this study, EID was more effective for the treatment of memory impairment and neurodegeneration of the hippocampus than WID. Also, the effect of EID was similar to CAF. It has been shown that CAF was estimated to be 4 mg/g in water extract and to be 5 mg/g in 70% methanol extract among ID [12]. According to these quantitative amounts of the compounds, CAF concentrations in ID extracted by water extract and 70% methanol extract were not different, and similar results are expected from EID and WID with the TMT-induced animal model. However, treatment with EID to TMT-induced rats enhanced more effectively learning and memory capability and increased neuronal protective effect compared to WID. In addition, EID showed a more improved behavioral performance, cognitive function and decreased neuronal malfunction than CAF, suggesting that CAF is not a major compound but one of the bioactive components in ID for protecting neurodegeneration. Further studies should be conducted to determine neuroprotective bioactive components in EID. This study has several limitations. Firstly, although the study observed the increased expression of ChAT and CREB, the precise molecular mechanisms through which EID and CAF exert their neuroprotective effects remain unclear. Further research is needed to elucidate these mechanisms in more detail. Secondly, the effects of EID and CAF were evaluated over a relatively short period. Long-term studies are necessary to assess the chronic effects on cognitive function and neuronal health. Such research will help determine the potential for sustained benefits and the safety of prolonged use. Third, in terms of statistical analysis, the current study utilized a relatively small sample size (6 to 8 rats per group) and these may reduce statistical power, and findings from these small sample may not be applicable to the clinical population, limiting the study’s external validity. In addition, our results derived from small samples may have wider confidence intervals, making it difficult to draw definitive conclusions of our study. This may limit the interpretation of the findings, and addressing these aspects in future studies could provide more robust insights for other researchers.

## 5. Conclusions

The administration of EID alleviated the TMT-induced memory impairment more effectively than WID, as demonstrated by performance on the MWM test. Also, the EID improved cognitive function and produced a neuroprotective effect by increasing CREB expression. Thus, EID may be a useful agent for preventing and protecting neurodegenerative diseases such as dementia and AD. It may be a good supplement for health care and be a good resource for drugs to prevent dementia and AD.

## Data Availability

The data used to support the findings of this study are available from the corresponding author upon request.
